# An Account of the Fatal Effects Produced by Attempting to Remove a Ganglion by Seton

**Published:** 1784

**Authors:** 


### II. *An account of the fatal effects produced by attempting to remove a Ganglion by Seton. Communicated in a letter to Dr.* Simmons, F. R. S. *By Mr.* William Deafe, *Surgeon to the United Hofpitals of St. Nicholas and St. Catharine at Dublin.*

THE following cafe, which I take the liberty of communicating to you, that it may be inferted, if you think proper, in the London Medical Journal, will, I hope, ferve as a ftriking inftance of the painful and fatal confequences that may follow an attempt to remove Ganglions by Seton.

In July 1781, a clergyman, aged 37, confulted me about a moveable Ganglion of the fize of a fmall nutmeg, fituated between the fore-finger and thumb of his right-hand, near the wrift. He was eager to have it removed, and had been advifed, for this purpofe, to have a Seton paffed through it as the beft and moft certain method; but as he was apparently a robuft healthy man, and the Ganglion was attended with no pain, I advifed him to confider it as a matter of no confequence, and not to meddle with it.

Four

Four months after this I was desired to visit him, and found him in a melancholy situation. A Seton had been passed through the Ganglion, and the consequences were, that the back of his hand had inflamed violently, that the Ganglion had rapidly and amazingly increased, and that the openings made by the Seton were filled with an ill-conditioned fungus, which sprung up as fast as it was removed, and was attended with frequent hæmorrhage and much pain.—In consultation it was agreed to remove this fungus by a free incision, which was done, and the metacarpal bones appeared bare and rough. Another opening was made through the Thenar, and a Seton passed through it, in order more effectually to prevent the growth of fungus. The bark was administered in large quantities, an opiate was given at night, and due attention was paid to the regimen of the patient. This method seemed to promise the most happy event. The fungus appeared to be entirely destroyed, a laudable suppuration took place, the swelling of the hand subsided, and the sores, in a short time, were so contracted as to indicate their speedy cicatrization. These favourable appearances, however, were not of long continuance; for after some time the fungus began gradually to riſe again, and any mode of keeping it down either by cauſtic, cutting, or preſſure, ſeemed to produce no permanent good effect, as it increaſed rapidly, and at length degenerated into the moſt frightful cancerous fungus I have ever ſeen. Every local application that has been recommended in ſimilar caſes was tried in this, but without ſucceſs; and internal remedies proved equally inefficacious. He took, for a conſiderable length of time, two ounces of bark, in ſubſtance, in the courſe of twenty-four hours, ſo that he took in the whole twenty-eight pounds of that medicine. The extract of hemlock had alſo a fair trial, but produced no apparent good effect.

When he had laboured under this complaint fifteen months, he was adviſed to undergo the amputation of his hand; but before he would conſent to ſubmit to this operation he choſe to have an account of his caſe tranſmitted to the Royal Academy of Surgery at Paris, that he might have their opinion of it: the reſult of which was, that the members of the Academy pronounced the fungus, not cancerous, but merely ſcorbutic. This deciſion, by the bye, ſhould make us extremely cautious in delivering our our ſentiments on ſimilar occaſions; without ſeeing the patient, as much depends on the general appearance of the ſores in caſes of this ſort. The Academy were of opinion, that the diſeaſe was entirely local and required only local treatment. For this purpoſe they adviſed that the fungus ſhould be taken down by means of Euphorbium, Savine, &c. and afterwards waſhed with ſalt water. If this method proved ineffectual recourſe was to be had to the actual cautery, from the application of which they ſeemed to expect the moſt deciſive advantages. To this mode of treatment the unhappy ſufferer ſubmitted, and during the ſpace of ſix weeks the fungus was almoſt every day burnt down with the cautery, but his complaint all the while continued to gain ground apace, ſo that being now diſappointed in all his expectations of relief from regular practitioners he had recourſe to quacks of every denomination. The arſenic plaiſter of Plunket was applied, and he was ſalivated for ſeven weeks. At length, after undergoing the operation of a variety of noſtrums, he again placed himſelf under my care. In conſultation it was much doubted whether amputation ſhould now be thought of, as the patient ſeemed to be in the laſt ſtage of a cancerous conconsumption. His limbs were swelled, and his whole habit was wasted by the repeated hæmorrhage from the fungus, which was now so increased in bulk as to weigh down his arm, and entirely cover the back of his hand. In short, after every return of hæmorrhage, it was apprehended that the next would put a period to his sufferings.

The hazard of the operation, and the little chance he had of its proving successful, being explained to him, the unfortunate man earnestly begged to be relieved from so hideous a load, even though he should die under the operation. I therefore yielded to his intreaties, and took off the hand a little above the wrist in November 1782, altho' there was a small indurated gland above the elbow.

On dissecting the hand immediately after I had taken it off, the fungus, on being cut, appeared to be extremely similar to the substance of the brain, and to arise from the metacarpal bones of the middle and fore fingers. These bones were in part dissolved, and the other bones of the hand were also in a morbid state.

No accident occurred during the amputation, but soon after it a colliquative diarrhœa came on, which seemed to be increased by opiates and astrinaſtringents, but was at length checked with draughts of fixed alkaline ſalt and lemon juice, ſwallowed in a ſtate of effervefcence. He afterwards took the bark, drank Seltzer water, and was allowed a liberal uſe of wine. The ſuppuration for ſome time was ichorous and bad, but he gained ſtrength daily. At the end of ſeven weeks the ſtump was completely cicatrized, and the indurated gland above the elbow had diſappeared. He went into the country, drank goats whey, bathed in the ſea, became very corpulent, and ſeemed to be in perfect health, but had ſomewhat of a ſallow, bloated appearance. He continued well till July 1783, when he began to complain of pains in his back, attended with rigidity. Theſe pains, as they increaſed, extended down his thighs and legs, and occaſioned him to ſleep ill at night. He grew feveriſh, his pulſe beat extremely quick, and his countenance acquired a ſhining yellowiſh red colour, an appearance which I have remarked to be characteriſtic of a cancerous habit. He now began to walk with difficulty. I took a ſmall quantity of blood from him, and found the texture of the craſſamentum extremely looſe, and the ſerum in too great quantity. He was very difficult to purge, and unfortunately was

 under under a conſtant neceſſity of taking medicines to procure the neceſſary diſcharges. Antimonials in a variety of forms were given, and the bark was again tried, as were all the medicines that are uſually preſcribed in rheumatic caſes. Bliſters were applied, and iſſues cut in his thighs, but all to no purpoſe. He was obliged to take to his bed in Auguſt, and never after quitted it.

It is difficult to form an idea of the conſtant and excruciating pain this poor man ſuffered. Opium, though given in large doſes, afforded him but little relief, and at laſt none at all. He generally laid on his back, fixed as it were to the bed, the leaſt motion occaſioning the moſt intenſe pain. As the diſeaſe advanced he complained of a difficulty of paſſing his urine, which was loaded with a viſcid mucus, and he once diſcharged an oblong calculus; but at laſt he voided his urine involuntarily, and ſometimes even his fæces, but the latter only rarely, when he had taken a purgative, which, as I have already mentioned, was required to be of the moſt active kind, otherwiſe it produced no effect.

During the whole courſe of the diſeaſe his pulſe was rapid, but his tongue was remarkably ſoft and florid. He was never delirious. Latterly he ſpit blood once or twice; his lower extremities ties became very œdematous, and his back was covered with eſchars, but theſe dropped off, and the ſores ſuppurated and healed kindly. Two months before his death his pains abated conſiderably. He died without pain, March 4, 1784, which was about two years and nine months from the time the ſeton was paſſed, and a year and four months from the time he underwent the amputation.

His body was opened a few hours after his death. The abdominal viſcera appeared to be perfectly ſound and of their natural colour, except the liver, which had a ſmall ſteatoma on its convex ſurface, but was in other reſpects healthy. The gall-bladder was rather fuller of yellow bile than it is generally found to be. The left kidney was enlarged, and on dividing it longitudinally much red gravel was found in its pelvis, and the ureter ſeemed much leſſened. The urinary bladder was contracted, and its coats uncommonly thickened, but no ſabulous concretions were obſerved in it.

On each ſide of the vertebræ lumborum the lumbar regions were rendered convex by a large cancerous depoſition, which elevated the pſoæ muſcles, and when the cellular inveſtitures, which were condenſed into a cyſt, were opened, the cancerous matter appeared in a large quantity, in colour and confiftence exactly refembling the fungus of the hand, and not unlike the fubftance of the brain. The whole weighed about five pounds, and when this was removed, the laft vertebra of the back, and the three firft of the loins were found to be in a foftened, eroded, and in fome parts a totally diffolved ftate. There appeared not the leaft mark of ichor, fanies, inflammation, or hardnefs of the foft parts, nor were the mefenteric glands at all affected. The matter feemed to have been really a cancerous exudation, and to be formed chiefly of coagulable lymph. This cancerous mafs feemed to poffefs a remarkable diffolving power, which was exerted wholly on the bones, and did not, as ufual in cafes of this fort, caufe any fchirrous hardnefs of the furrounding foft parts.

This cafe may furnifh many practical inferences. In the firft place, it may ferve to fhew the great danger of developing in certain conftitutions a latent virus. The patient was certainly in the enjoyment of full health when I firft faw him, and probably might have long continued fo, if recourfe had not been had to the feton. I have obferved, that by far the greater number of cancers happen to thofe who have been remarkably healthy before, but who have had at the ſame time an irritable nervous ſyſtem, fully characterized by their general conduct thro' life. The religious orders in Roman catholic countries, among whom cancer is a very frequent complaint, are a ſtrong proof of this, and in ſuch conſtitutions we ſhould be extremely cautious how we operate, except in the moſt preſſing circumſtances.

Secondly, in all caſes of ganglions of cancerous tendency, extirpation will, I believe, be found to be far preferable to any other mode of treatment, as a cancerous fungus ſoon imparts to the general maſs of humours the cancerous virus, ſo as to render the good effects of any ſurgical operation extremely doubtful.

Thirdly, from a careful inquiry into the origin of cancers, I believe the diſeaſe, in the beginning, is ſeldom humoral, but rather to be imputed to a morbid degree of irritability in the nervous ſyſtem. The phenomena of the diſeaſe and the medicines which have produced any good effect in it, being of the claſs of ſedatives, ſeem to me to be very corroborating proofs of this opinion.

Fourthly, we may infer, that cancerous matter does not always fall on the glandular ſyſtem; for for in the preceding caſe, and in others which I have ſeen, it ſeems to have been formed rather by exudation, no glands having appeared to be in any degree enlarged.—How this humor was ſtrained from the circulating maſs, and depoſited in the lumbar region, our limited knowledge of the laws of ſecretion and excretion will not permit us even to hazard a conjecture.

I ſhall conclude this prolix detail with mentioning a very ſingular circumſtance that conſtantly attends a morbid degree of nervous erethiſm, which is, that although every other part of the human body ſeems to be tremblingly alive, the inteſtinal canal is particularly exempt from this irritable ſtate, as we find patients of this conſtitution extremely hard either to vomit or purge, and this is in general the caſe with all patients labouring under cancer.

Dublin,
April 24, 1784.

